# Dear *BJPsych Bulletin*…

**DOI:** 10.1192/bjb.2018.57

**Published:** 2018-10

**Authors:** Norman Poole

**Affiliations:** 1 South West London and St George's Mental Health NHS Trust

## Abstract

The editor thanks the outgoing Correspondence Editor Greg Shields and welcomes Dr William Badenhorst as his successor. He reflects upon his own inadequacy as a letter writer in the hope it encourages others to be better.

**Declaration of interest:**

None.

I must confess that I do not often write letters. As a child I had to be cajoled and then threatened into composing thank you letters at Christmas. I assumed (wrongly, it turned out) that a mumbled ‘Thanks’ was quite sufficient. If only I'd known Heidegger's arguments for the supremacy of the spoken word at the age of eight, my life would have been easier in so many ways. I'm still no better at postcards, notes of condolence, or work emails, and even texts feel just a little too much effort. It should come as no surprise that as a medical student I did not attempt to submit letters to the *BMJ* to gain an ‘easy’ first publication. Nor do I Tweet.

All this avoidance of letter writing would not appear to be good preparation for a career in psychiatry, however, and I am always immensely impressed by those who do put pen to paper. Freud was of course a prodigious writer of letters;^1^ these are worth a read for the light they shed on the man's foibles as much as the development of his ideas. His letter to the concerned mother of a young gay man,^2^ in which he assures her that homosexuality is ‘nothing to be ashamed of’ and its persecution ‘a great injustice’, exemplifies the qualities of a good letter: crafted, lucid and brief, with a hint of wit. Not everyone can aspire to such prose, but the correspondence section of a journal is often its most lively and combative. Recent notable examples within these pages include the exchange between Sameer Jauhar and Allan Young^3^ and Joanna Moncrief,^4^ following her typically provocative paper in our *Against the Stream* series. I was also struck by Martin Plöderl and Clemens Fartacek's admirably brief account of complexity theory in risk assessment in April's edition.^5^ A well-written rebuttal can influence minds as much as any original paper, and quicker too.

Notwithstanding some admiration for the scribblers’ art, you can imagine my state of mind when our correspondence editor Greg Shields handed in his notice just a few weeks into my editorship. (A temporal relationship need not imply causality!) Greg has worked on the section for 2 years, but a first consultant post means he will now be frantically trying to keep on top of his own correspondence. I'd like to take this opportunity to thank Greg for his sterling work over the past couple of years and wish him all the best in all future endeavours. And I'm able to say this sincerely because in between his tendering his notice and now, we have interviewed for a new correspondence editor and, from a selection of three excellent candidates, appointed William Badenhorst as Greg's successor. William is certified in both general adult psychiatry and medical psychotherapy so is trained to efficiently process and analyse the many letters sent to the *BJPsych Bulletin*. He promises a fair but firm approach to the publication of eLetters online and in the correspondence section of the journal. Sadly, not every submission realises each of the qualities listed above, and some none at all. Don't let this put you off writing your own letter to the editor, however. Re-read this editorial and remind yourself, ‘I could do better than that’, then get going. Just don't expect a reply.

## About the author

Dr Norman Poole is Editor of the *BJPsych Bulletin* and Consultant Neuropsychiatrist at St George's Hospital, South West London and St George's Mental Health NHS Trust, UK.
